# Genetic mechanisms underlying temperature-mediated changes in flower development

**DOI:** 10.1093/jxb/erag306

**Published:** 2026-06-20

**Authors:** Roosa A E Laitinen, Paula Elomaa

**Affiliations:** Department of Organismal and Evolutionary Biology, Viikki Plant Science Centre, 00014, University of Helsinki, Finland; Department of Agricultural Sciences, Viikki Plant Science Centre, 00014, University of Helsinki, Finland; University College Dublin, Ireland

**Keywords:** Adaptation, evolution, flower development, phenotypic plasticity, temperature

## Abstract

Flower morphologies are remarkably diverse in nature, reflecting their specialized role in plant reproduction. In the context of climate change, understanding the impact of temperature on flower development is essential for predicting plant adaptation to future climates, and improving crop resilience. The genetic basis of flower development is well understood, but less is known of the impact of environmental variation on floral traits and the genetic mechanisms mediating such responses. This gap in knowledge may stem from the longstanding view that flower development is buffered against environmental influences due to its essential role in fitness. However, growing evidence indicates that floral traits can be environmentally plastic, adjusting in response to factors such as temperature, light, and water availability. This review gathers current knowledge on the temperature effects of floral traits and explores the genes and mechanisms underlying the temperature-mediated changes in flower development. We highlight trait-specific responses and consider the adaptive consequences of temperature-mediated trait variation.

## Teaser

This review brings together current knowledge on changing temperatures on genetic regulation of flower development traits and their plasticity, and their implications for plant adaptation and resilience, highlighting the importance of understanding environmental impacts on flowers.

## Introduction

Flower development, from the establishment of organ identities to the effects on organ numbers, size, and shape, has been extensively studied in many different species. These studies have revealed several genes and genetic mechanisms governing the enormous morphological diversity across the plant kingdom (Theißen *et al.*, 2016; Moyroud and Glover, 2017; Chen *et al.*, 2018; Bowman and Moyroud, 2024). Yet, despite the detailed knowledge of the genetic basis of flower development, we still know surprisingly little about the natural variation within populations in genes underlying flower development. Bridging this gap requires not only an understanding of the existing genetic variation in flower developmental genes, but also fundamental knowledge of how environmental change influences their regulation and expression.

Temperature is one of the key factors projected to change in the future, with global temperatures predicted to increase by 2–5 °C by the year 2050. While the temperature responses of plant growth and development, known as thermomorphogenesis, are well documented in vegetative traits and in flowering time (Quint *et al.*, 2016; Ibañez *et al.*, 2017; Casal and Balasubramanian, 2019; Delker *et al.*, 2022), our understanding of the mechanisms underlying temperature-driven changes in flower development are still limited.

**Fig. 1.**
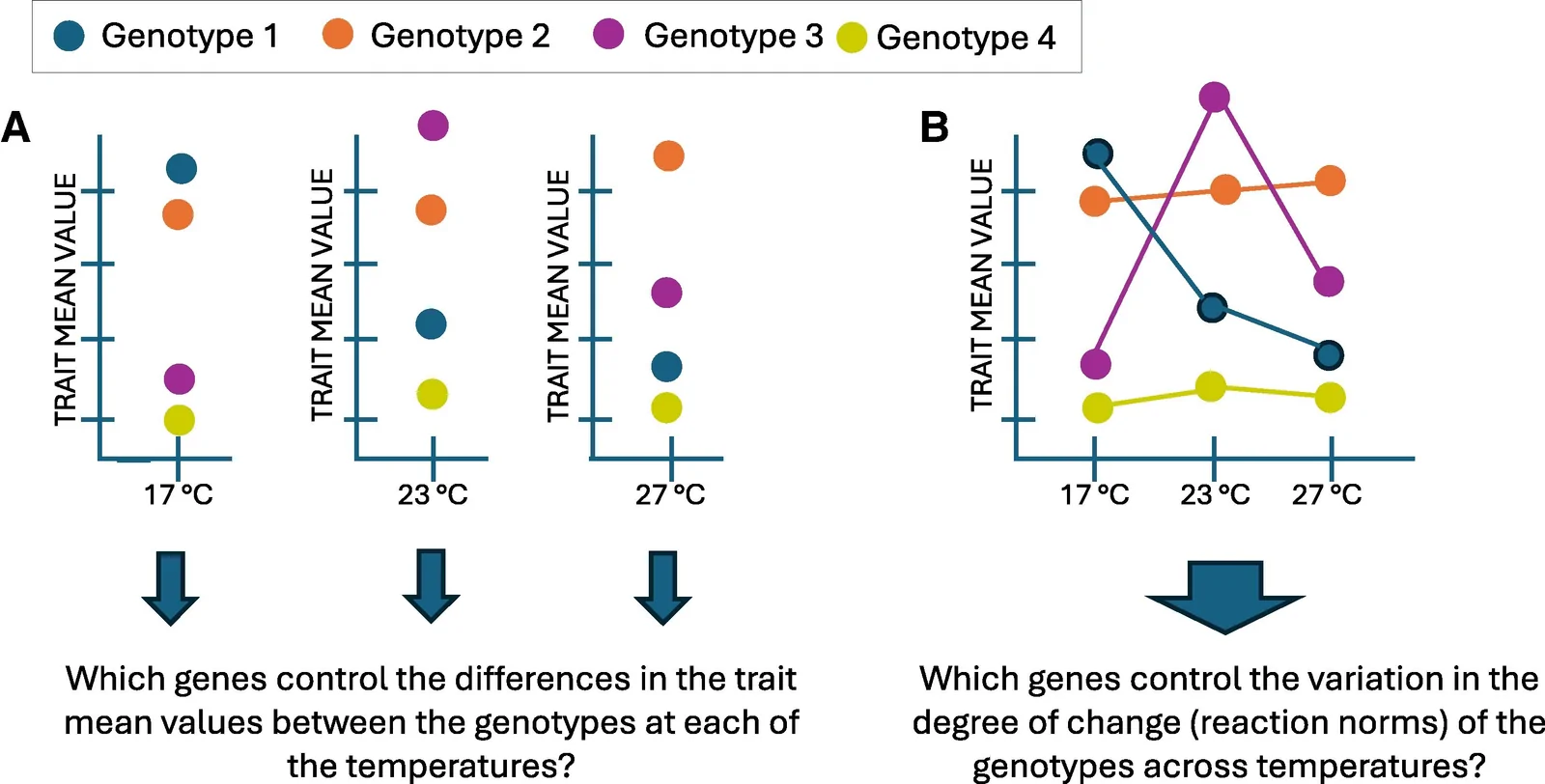
Genetic basis for the trait mean values and trait plasticity can be independent of each other. This schematic graph demonstrates that if we compare the mean values (A) of genotype 2 and genotype 4, they show differences and different genetic basis. However, they have similar reaction norms (B) and show same amount of plasticity and therefore their ability to maintain stable could be controlled by the same genes. Independent genetic control of trait mean value and trait plasticity to environments would allow these traits to not only evolve, but also to be engineered independent of each other.

To understand plant responses to temperature, we need to identify both the genes controlling the mean value of a given trait at different temperatures as well as the genes controlling temperature-mediated plasticity (Fig. 1). Temperature-mediated phenotypic plasticity, which can be measured at the level of an individual or a population, is defined as the degree of change of a trait in a single genotype in response to different temperatures (Fig. 1). Flower development has been thought to be highly buffered against environmental perturbations, including temperature. This may be explained by the theory of canalization, which proposes that key developmental traits, such as those associated with flower development and closely linked to plant fitness, remain stable despite environmental fluctuations (Waddington, 1942). Yet, in contrast to canalization theory, studies that will be reviewed here, have challenged the view of strictly canalized development by demonstrating that environmental factors such as temperature can significantly influence genes controlling flower development. Given the central role of floral traits in reproductive success, even subtle temperature-induced changes can have significant adaptive consequences.

The current knowledge of temperature-driven changes in flower development is reviewed here. We will first review literature related to mechanistic understanding of temperature on regulation of genes underlying flower developmental traits including flower organ identity, flower organ numbers, and inflorescence architecture. In the second part, we will focus on the current knowledge of temperature-mediated plasticity genes in flower developmental traits, and in the third part, we will discuss the adaptive significance of the temperature-regulated flower developmental genes.

### Impact of temperature on the genetic regulation of flower development

In this first part, we focus on selected examples of the influence of temperature on regulation of genes underlying flower organ development, petal number variation, and inflorescence architecture. These studies utilize mutants to understand the role of given genes to the control of mean values of floral traits in plants grown under different temperature conditions.

### Temperature influences flower organ development through regulation of homeotic gene functions

Flower morphology is highly diverse across flowering plants, yet it remains strongly conserved within a species to ensure successful reproduction. While species in the ANA-grade angiosperms (*Amborellales*, *Nymphaeales*, *Austrobaileyales*), magnoliids, and some non-core eudicot lineages such as *Ranunculales* show more lability in organ numbers and their arrangements, the whorled floral ground plan in monocots and core eudicots (*Pentapetalae*) is highly stable (Endress, 2010, 2011). Moreover, genetic regulation of flower organ identity in distinct lineages is remarkably conserved and defined by the canonical ABCE model (Bowman *et al.*, 1991; Coen and Meyerowitz, 1991; Pelaz *et al.*, 2000; Honma and Goto, 2001).

The ABCE activities mostly correspond to the combinatorial action of MADS-box transcription factors, except for APETALA2, a transcription factor of the AP2/EREBP family (Jofuku *et al.*, 1994). By forming distinct tetrameric complexes, they specify organ identities in concentric rings or whorls; the activity of A class proteins define the development of protective sepals in the outermost whorl, A and B functions together are required for petal identity, B and C for stamen and C for carpel development while the E function contributes to all whorls (Smyth *et al.*, 1990; Bowman *et al.*, 1991; Pelaz *et al.*, 2000; Honma and Goto, 2001; Theissen and Saedler, 2001; Theißen *et al.*, 2016; Bowman and Moyroud, 2024). Although stable organ identities are essential for successful reproduction, there is evidence indicating that organ identity genes respond to changes in ambient temperature. A critical first step towards understanding the influence of temperature on flower development is to identify organ identity genes that show temperature-dependent regulation. These temperature-mediated changes in gene function may impact reproduction and consequently fitness.

The earliest examples of the role of temperature on organ identity regulation emerge from the temperature-sensitive mutants identified from *Antirrhinum majus* and *Arabidopsis thaliana* models. The *A. majus* mutant phenotype of the B-class MADS-box gene *DEFICIENS* (*DEF*) *def-101*, caused by a single amino acid deletion, is highly dependent on ambient temperature (Zachgo *et al.*, 1995). The *def-101* mutant flowers display severe abnormalities when grown at a higher ambient temperature of 26 °C resulting in sepaloid petals, carpelloid stamens, and a modified central gynoecium with multiple locules. However, when grown at a low ambient temperature of 15 °C, the flowers largely resemble those of the wild type. It was proposed that the higher temperature contributes to unstable protein–protein interactions or DNA-binding of the DEF-101 protein necessary for its function (Zachgo *et al.*, 1995).

In addition, in *A. thaliana*, elevated temperature intensifies floral defects in the *ap3-1* mutant of the B-class *APETALA3* (*AP3*) gene (Sablowski and Meyerowitz, 1998). In the *ap3-1* mutant, the organ identity abnormalities in petals and stamens were more pronounced when the mutant was grown at 28 °C compared to 16 °C (Sablowski and Meyerowitz, 1998). In this case, the phenotype was shown to arise from a temperature-dependent splicing defect further disrupting the autoregulatory loop involving the B-class proteins AP3 and PISTILLATA (PI). Although representing examples of variability in homeotic mutant phenotypes, these examples imply that temperature may modify key MADS-box gene functions and/or the regulatory networks they are involved in.

In rose (*Rosa* sp.), ambient temperature change (16–28 °C) affects petal and stamen development through epigenetic regulation of a C-class *AGAMOUS* (*AG*) gene. Low ambient temperature significantly increases petal numbers through homeotic change of stamens into petals (Ma *et al.*, 2015; Lu *et al.*, 2024). This change has been associated with a reduced expression level of *AG* in the stamen whorl. This change has been associated with a reduced expression level of AG in the stamen whorl caused by enhanced methylation of the *AG* promoter under lower ambient temperature conditions (Ma *et al.*, 2015). A recent study in *Rosa chinensis* identified two short interstitial telomere motifs (telo-boxes) in the second intron of the *RcAG* gene (Lu *et al.*, 2024). At lower temperature, these telo-boxes were shown to regulate *RcAG* expression by recruiting a transcriptional repressor complex. This intron-based mechanism allows the plants to perceive the temperature signals that consequently regulate flower organ development.

In the cereal crops rice and barley, the *SEPALLATA3*-like (*SEP3*) E-class *MADS8* genes show a conserved function in regulating temperature-dependent female organ development (Shen *et al.*, 2023). At 28 °C, the knockout mutants exhibited multiple carpels, and the ovules were replaced by indeterminate carpel-like structures, while they did not show any visible changes when grown at 15 °C. Intriguingly, the barley MADS8 gene, *HvMADS8*, showed increased numbers of downstream target genes at higher temperatures. This suggests that temperature alters the binding properties of the protein, potentially through conformational changes, while the protein–protein interactions with other floral MADS proteins were not affected. Similar conversion of ovules into carpelloid structures under high ambient temperatures was also observed in *A. thaliana* (Modrusan *et al.*, 1994), suggesting that the temperature dependency of female organ development is common across plant species.

### Mechanisms buffering the effect of homeotic mutations due to changing temperatures

In addition to the examples of temperature-regulated homeotic genes resulting in changes in flower development, there are examples of mechanisms that prevent homeotic changes in flowers in response to different ambient temperatures. Recently, the plant specific Class II TEOSINTE BRANCHED 1/CYCLOIDEA/PCF (TCP) transcription factors were shown to redundantly protect ovule identity and development at high ambient temperatures in *A. thaliana* (Lan *et al.*, 2023). The homeotic conversion of ovules to carpelloid structures in a duodecuple null mutant *tcp2/3/4/5/10/13/17/24/1/12/18/16* (*tcpDUO*) was inhibited by complementation with *TCP4*. At the mechanistic level, under higher ambient temperature, TCP4 was shown to stabilize the BELL (BEL1) protein. BEL1 in turn is a key regulator of ovule identity, that represses the activity of the MADS-box protein quartets composed of C- and E-class AG and SEP proteins, known to specify carpel identity (Ray *et al.*, 1994).

In addition to *A. thaliana*, genes buffering changes in flower organ identities have also been identified in rice. The rice gene *EXTRA GLUME 1* (*OsEG1*) has been shown to promote floral robustness against temperature fluctuations upstream of floral organ identity genes (Zhang *et al.*, 2016). Rice *eg1* mutants exhibit a higher frequency of floral abnormalities when grown at high-temperature conditions. OsEG1 was shown to promote the expression of *OsMADS1* and *OsMADS6*, and all collectively control the differentiation of three inner organ whorls, maintaining stable flower development. From these, *OsMADS1* is a *SEP*-like MADS-box gene affecting lemma/palea identity and *OsMADS6* is an *AGL6*-like gene that regulates floral determinacy, in addition to the development of all four whorls of floral organs in rice.

High ambient temperature promotes the expression of *OsEG1* and increases the stability and lipase activity of the protein encoded by it (Zhang *et al.*, 2016). OsEG1 is thought to function through lipid metabolism, but it also influences jasmonic acid (JA) biosynthesis and signalling, thereby linking temperature perception to the hormonal and metabolic regulation of floral organ identity (Zhang *et al.*, 2016). In addition to *OsEG1*, mutations in rice *BARENTSZ* (*OsBTZ*) genes lead to increased developmental abnormalities, including disrupted stamen and carpel functionality, butat low temperatures (Li *et al.*, 2024). The OsBTZ protein physically interacts with the SEP-like OsMADS7/8, indicating that temperature-responsive protein–protein interactions among MADS-box factors are critical for maintaining floral patterning when buffering against suboptimal temperatures (Li *et al.*, 2024).

### Temperature-mediated petal number variation

**Fig. 2.**
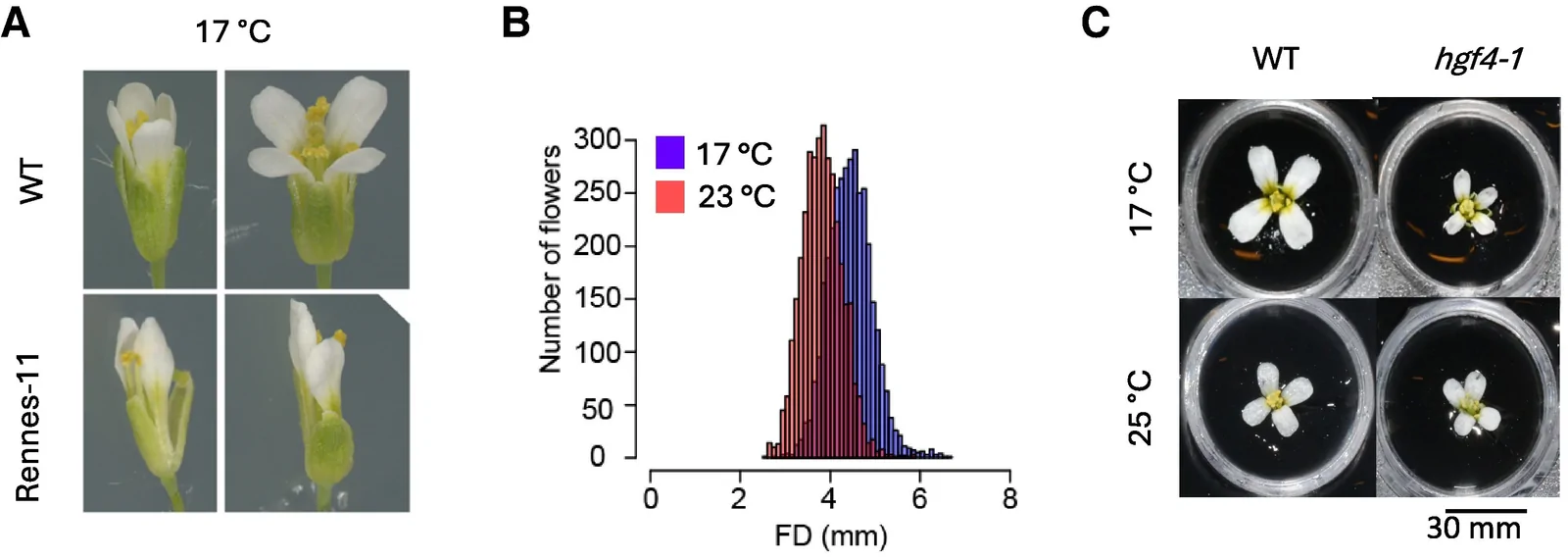
Examples of the temperature-mediated responses in *A. thaliana* flowers. (A) *A. thaliana* accession Rennes-11 shows reduced number of petals when grown at 17 °C after vernalization (photo credit: Teng Zhang, University of Helsinki). (B) Distribution of the mean flower sizes of 290 *A. thaliana* accessions grown at 17 °C and 23 °C shows a clear shift in the mean flower sizes towards bigger flowers at lower ambient temperature (Wiszniewski *et al.*, 2022). (C) In the spliceosome mutant *hgf4-1* flowers are significantly smaller than the wild type when grown at 17 °C, resulting in reducing flower size plasticity (photo credit: Gregory Andreou-Huotari, University of Helsinki). FD = flower diameter.

Flower organ numbers are usually highly invariant within the species and not affected by environmental perturbations. While genetic regulation of petal numbers is relatively well-studied, understanding of the temperature effects is limited (Wang *et al.*, 2024). In the *A. thaliana* Rennes-11 (Rennes, France) accession, petal number is reduced in response to vernalization and lower ambient temperature (Fig. 2A). Yet, the genetic control of the low temperature-regulated variation is unknown.

*Cardamine hirsuta*, a close relative of *A. thaliana* in the *Brassicaceae* family, provides a model system to understand petal number variation in response to temperature. In *C. hirsuta*, petal numbers may vary from zero to four in flowers of a single plant (Monniaux *et al.*, 2016; Pieper *et al.*, 2016; McKim *et al.*, 2017). Intriguingly, spring flowering *C. hirsuta* plants were shown to generate more petals than summer flowering plants. The most significant increase in petal numbers was associated with low ambient temperature during the spring that was shown to extend the time of flower meristem maturation. This in turn resulted in larger meristems. The larger meristem size generated wide, and more slowly growing spaces between the emerging sepals which then could occupy more petals (Pieper *et al.*, 2016; McKim *et al.*, 2017). Notably, petal numbers were also affected by endogenous cues such as plant age, plant hormone gibberellic acid as well as photoperiod, light quality, and vernalization, while the numbers of other floral organs were not affected (McKim *et al.*, 2017).

Petal number in *C. hirsuta* also shows natural genetic variation that has been associated with at least 15 quantitative trait loci (QTLs) (Pieper *et al.*, 2016). A conserved floral regulator, *APETALA1* (*AP1*) was shown to have functionally diverged between *A. thaliana* and *C. hirsuta*, and to contribute to lack of petal number robustness in *C. hirsuta* (Monniaux *et al.*, 2018). The role of *AP1* in *C. hirsuta* was associated with the loss of epistasis of *AP1* over the QTLs that cause petal number to vary (Monniaux *et al.*, 2018; Rambaud-Lavigne *et al.*, 2025). In addition to *C. hirsuta*, *AP1*-like genes regulate temperature-dependent petal number variation in tulip (Leeggangers *et al.*, 2017) and in the ornamental tree species *Osmanthus fragrans* (Liu *et al.*, 2023).

A recent study also focused on altered patterning of *C. hirsuta* floral meristems (Rambaud-Lavigne *et al.*, 2025). The organ boundary regulators CUP-SHAPED COTYLEDON (CUC) 1 and 2 were shown to regulate auxin accumulation in the inter-sepal regions specifying the positions of initiating petals (Rambaud-Lavigne *et al.*, 2025). This contrasts with *A. thaliana* where sepal and petal patterning occurs in distinct whorls. However, the role of ambient temperature in increasing petal numbers was independent of CUC activity, suggesting the involvement of additional factors in organ patterning (Rambaud-Lavigne *et al.*, 2025). Yet, it is still unclear how this major developmental shift results in loss of robustness in petal numbers at the mechanistic level.

### Inflorescence architecture is affected by higher temperature

**Fig. 3.**
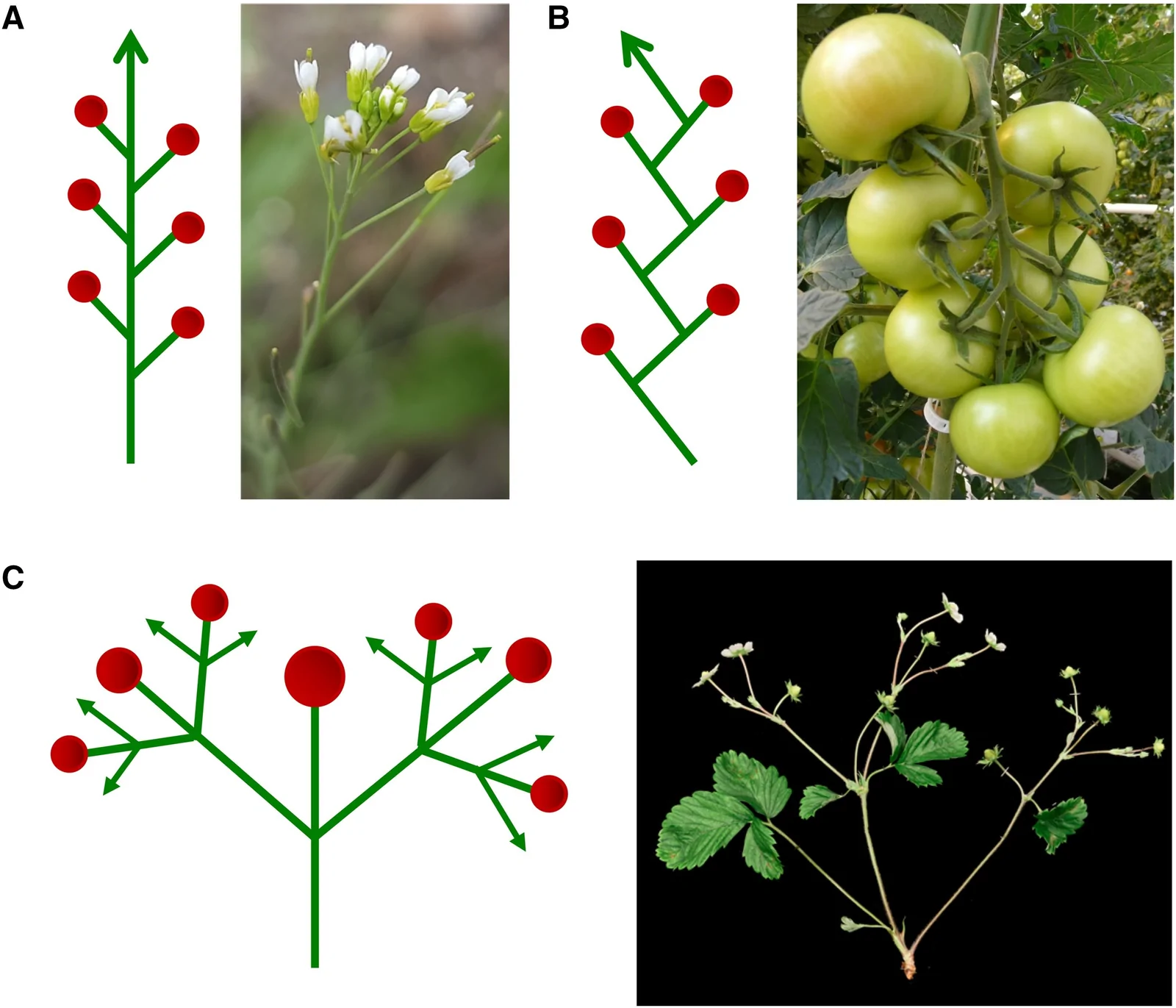
Examples of diversity of floral displays in inflorescences. (A) A basic monopodial raceme as in *Arabidopsis thaliana* grows continuously and generates flowers in a sequential manner from the main axis (photo credit: Elli Kotka, University of Helsinki). (B) In sympodial growth, the main axis terminates into a flower, but the growth continues from a secondary meristem creating a zig-zag pattern. In tomato inflorescences, the flowers, and consequently the fruits, are formed in zig-zag-like trusses. (C) The level of inflorescence branching in compound inflorescences such as thyrses in the wild woodland strawberry (*Fragaria vesca*) may vary and ultimately determine final flower number affecting berry yield (photo credit: Sergei Lembinen, University of Helsinki).

The complexity of floral displays is determined by the final numbers of flowers and their arrangement in space and time within inflorescences. Thereby, inflorescence architecture is ultimately defined by patterning of meristems that generate floral primordia. It is well known that modifications in the timing of meristem transitions, in meristem maturation and termination involving conserved floral regulators, dramatically alter inflorescence architecture as demonstrated in tomato (Park *et al.*, 2012; Soyk *et al.*, 2017) and woodland strawberry (Lembinen *et al.*, 2023). Consequently, inflorescence architecture may vary from a single flower to highly complex structures with multiple branches and large numbers of flowers (Fig. 3).

A key factor affecting final inflorescence architecture is the level of branching. Ambient temperature has been shown to modulate inflorescence branching in the sympodial species tomato (*Solanum lycopersicum*) (Sun *et al.*, 2024). Tomato *qMULTIPLE INFLORESCENCE BRANCH 2* (*qMIB2*) was shown to positively regulate inflorescence branching in response to high ambient temperature, consequently decreasing the weight of individual fruits. The non-functional *qMIB2* alleles in natural populations, however, were less sensitive to high temperature and produced fewer branches and fruits while fruit size (weight) was more stable (Sun *et al.*, 2024). Thereby, Sun *et al.* (2024) proposed that the *qMIB2* locus might have been selected during tomato domestication to maintain uniform fruit size ensuring higher or more stable yields at high ambient temperatures. *qMIB2* encodes a basic helix-loop-helix (bHLH) transcription factor and is very similar to the *A. thaliana* gene *SPATULA*, that regulates seed germination and vegetative growth in response to cold stress. However, in contrast to *SPATULA* in *A. thaliana*, *qMIB2* accumulates in response to high temperature.

In wheat and barley, high ambient temperatures (>25 °C) have been shown to inhibit inflorescence and spikelet development during early reproductive stages (Porter and Gawith, 1999; Ejaz and von Korff, 2017). Li *et al.* (2021) showed that in barley a SEP-like MADS-box protein HvMADS1 is required to maintain unbranched spike architecture at high ambient temperatures. In the *hvm1* mutant, increasing ambient temperature induced formation of ectopic spikelet-like organs and enhanced branching from ectopic meristems. This enhanced branching of the inflorescences occurred in a dosage dependent manner. HvMADS1 was shown to regulate the thermal transcriptome through increased binding to target A-tract CArG-motifs whose conformation changes with temperature. A cytokinin oxidase encoding the gene *HvCKX3* was identified among the targets indicating changes in cytokinin homeostasis in the meristem at high temperatures. At control temperatures, *HvCKX3* was shown to regulate spike determinacy and to prevent branching.

In rice, THERMOSENSITIVE BARREN PANICLE (TAP) secures normal inflorescence (panicle) and spikelet development under high ambient temperature (Zhang *et al.*, 2023). The panicles of the *tap-1* mutant were like those of the wild type at 22 °C and 28 °C, while at 30 °C they showed decreased numbers of secondary branches and spikelets, as well as floral organ defects due to defects in meristem functions. TAP belongs to the transposase-derived FAR1 RELATED SEQUENCE (FRS) protein family. It was shown to promote the expression of *OsYABBY3/4/5* genes as well as physically interact with OsYAB4/5 proteins, known regulators of panicle and spikelet development. Zhang *et al.* (2023) further discussed the potential involvement of TAP and OsYABs in recruiting chromatin remodelling factors to regulate temperature-sensitive changes in chromatin conformation. These examples highlight that high temperature (apart from heat stress) may affect plant reproductive development, and consequently yield, by affecting inflorescence properties. This area of research, although poorly studied, is key to developing thermally resistant, climate-smart crop cultivars (Jacott and Boden, 2020).

### Genes underlying temperature-mediated plasticity of flowers

The studies using mutants, summarized in the preceding section, focused on understanding genes that control the mean values of traits when plants are grown at different temperatures. In this second part, we focus on current knowledge on temperature-mediated plasticity genes, which are defined as genes that regulate the degree of change in flower development traits in response to temperature variation. Recent studies have shown that the genes underlying the mean value of a trait and those controlling its plasticity may be genetically independent (Laitinen and Nikoloski, 2019; Duarte *et al.*, 2021; Wiszniewski *et al.*, 2022; Fig. 1). Independent genetic regulation would allow trait plasticity itself to evolve through natural selection independently of the trait mean value (Schlichting, 1986; Scheiner, 1993). Therefore, exploring the genetic factors and molecular mechanisms underlying flower development influenced by temperature is critical for predicting plant adaptation and enhancing crop resilience.

The genetic basis controlling the degree of change is evident only at the population level and is referred to as genotype-by-environment (G×E) interaction (Fig. 1). There are only a few examples in which the G×E interaction across different temperatures for flowers has been reported. In the *extra glumes 1* (*eg1*) mutant in rice that promotes floral robustness against temperature, from the analysed traits, only the long and empty bract-like glumes showed G×E interaction, while in other floral traits G×E interaction was largely missing (Zhang *et al.*, 2016). In rose, flower size showed G×E interaction in response to high temperature, implying a genetic basis for temperature-mediated flower size plasticity (Liang *et al.*, 2017). Yet, knowledge of temperature-responsive genes controlling G×E interaction in floral traits is limited to *A. thaliana*.

### The *MAF2-5* gene cluster underlies temperature-mediated flower size plasticity

In *A. thaliana*, natural variation across 290 world-wide accessions showed that only a few degrees of change in temperature influenced flower size. On average flowers were larger when grown at 17 °C in comparison to 23 °C (Wiszniewski *et al.*, 2022; Fig. 2B). The presence of G×E interaction for flower size across the two temperatures indicated a genetic basis for temperature-mediated flower size plasticity further implying that selection could act on plasticity.

Genome-wide association analysis was used to identify genes controlling the G×E interaction of flower size. From these the *MADS AFFECTING FLOWERING 2-5* (*MAF2–5*) gene cluster on chromosome 5 was validated by mutants to be causal for temperature-mediated flower size plasticity (Wiszniewski *et al.*, 2022). The mutants of the *MAF2-5* gene cluster showed reduced plasticity as indicated by non-altered flower size at 17 °C in comparison to 23 °C, while in the wild type the flowers were significantly larger at 17 °C. This underscores the contribution of temperature-responsive flowering-time regulators to petal growth.

### Role of alternative splicing in temperature-mediated flower size plasticity

Alternative splicing is a post-transcriptional process that enables the generation of multiple transcript isoforms from a single gene, allowing plants to rapidly adapt to environmental cues. It is mediated by the spliceosome, a complex composed of five small nuclear ribonucleoprotein particles (U1, U2, U4, U5, and U6). While temperature is known to induce alternative splicing in hundreds of genes in leaves, temperature-induced alternative splicing in flowers has not been systematically studied. In the case of *MAF2* and *MAF3*, alternative splicing is known to regulate vernalization dependent flowering time at lower ambient temperatures (Rosloski *et al.*, 2013; Airoldi *et al.*, 2015), and recently both *MAF2* and *MAF3* were shown to undergo temperature-dependent alternative splicing in flowers (Andreou-Huotari *et al.*, 2026), indicating that alternative splicing could be an underlying mechanism mediating flower size plasticity in response to temperature.

Further supporting the role of alternative splicing in controlling flower size plasticity, reduced plasticity of flower size in response to temperature in a mutant of the core spliceosome factor *SmF* was recently associated with different splicing patterns of *MAF2* and *MAF3* (Andreou-Huotari *et al.*, 2026). In contrast to the *maf2*, *maf3*, *and maf4* mutants, in which petals were smaller at higher temperatures, the *SmF* mutant *hgf4-1* did not exhibit a reduction in petal size under elevated temperature (Fig. 2C). Altogether, these findings indicate that, in addition to the *MAF2–5* gene cluster being identified to control temperature-mediated plasticity of flower size, the degree of this plasticity may be modulated by alternative splicing. These findings suggest that alternative splicing may play a broader role in flowers, contributing to floral trait plasticity facilitating rapid environmental responses. It is intriguing to speculate that it also contributes to rapid adaptation of flower size in response to future environmental changes.

### Adaptive significance of temperature-mediated changes in flower development

In the third part of the review, we focus on evaluating the potential adaptive consequences of temperature-mediated changes in flower development. In natural populations, floral traits are closely linked to plant reproductive strategy. In outcrossing species, large and showy flowers enhance pollinator attraction, and flower size is typically maintained at a relatively stable optimum by strong positive selection (Stearns and Kawecki, 1994; Lempe *et al.*, 2013). In contrast, self-fertilizing species, such as *A. thaliana*, exhibit reduced flower size and increased number of flowers in comparison to their ancestral forms, likely reflecting selection to save resources for other, more direct, fitness-related traits (Worley and Barrett, 2000; Snell and Aarssen, 2005).

### Temperature-mediated changes in flowers can impact pollination

Around 90% of flowering plants depend on animal pollinators for reproduction (Tong *et al.*, 2023), and temperature influences both floral traits and activity levels of pollinator communities. Floral temperature itself can function as a signal: some flowers generate heat, producing thermal cues that guide pollinators (van der Kooi *et al.*, 2019; Harrap *et al.*, 2020), and recent evidence suggests that the temperature differential between a flower and the surrounding air can affect visitation rates (Koski *et al.*, 2026).

Furthermore, the reported temperature-induced variation in petal numbers in *Cardamine* was proposed to provide flexibility in mating under diverse environmental conditions (McKim *et al.*, 2017). Petal growth and opening are required for the flower buds to open. Consequently, low petal number at warmer temperatures results in reduced flower opening compared to higher petal numbers. Therefore, at high temperatures and under a high risk of dehydration, low petal numbers would not only reduce dehydration but also favour self-pollination of unopened flower buds. In contrast, higher petal number facilitates flower opening and outcrossing under cooler temperatures (McKim *et al.*, 2017). As reviewed above, temperature-driven shifts in floral morphology, phenology, or thermal properties may reverberate through plant–pollinator interactions, with cascading effects on reproductive success, population dynamics, and community structure, and this impacts ecosystem diversity.

### Temperature-driven homeotic changes can affect fitness

The adaptive significance of any trait variation is the outcome of genetic factors, environmental conditions, and G×E interactions. Considering that many homeotic mutants impair reproductive development and thereby play a role in fitness, temperature-driven induction of homeotic changes in future plant populations may have detrimental consequences. The change in homeotic gene expression controlling flower organ identities by suboptimal temperatures could disrupt normal development of reproductive organs, affecting pollen viability and ovule formation. In *A. thaliana*, it has been shown that there is natural variation in the sensitivity of reproductive organs to temperature, with Bur-0 accessions being highly sensitive to high temperature resulting in strongly reduced fitness (Huang *et al.*, 2014). In this context, temperature-induced changes in floral structure, such as altered organ arrangement or reduced complexity that maintain at least partial fertility, may be beneficial.

### Natural variation in temperature-induced genes underlying flower development

Existing variation in temperature-regulated genes controlling floral traits may result in phenotypic diversity within populations and have potential adaptive consequences. To date, the examples of genetic variation underlying temperature-regulated flower development in natural populations is scarce. Unlike many of the other developmental regulators, the *A. thaliana MAF2-5* gene cluster, underlying temperature-mediated flower size plasticity, is known to be highly variable among *A. thaliana* accessions (Theißen *et al.*, 2018). The variation could be the outcome of the role of the gene cluster in facilitating rapid adaptation in flowering time and further extended to regulation of flower size.

*Capsella bursa-pastoris* (shepherd’s purse), a close relative of *A. thaliana* and a worldwide distributed invasive weed, is a rare example in which a homeotic gene shows variation in nature, and thereby this species is used as an evolutionary model (Hameister *et al.*, 2009). The homeotic variant *Stamenoid petals* (*Spe*) shows conversion of petals to an additional whorl of stamens. The variant recently originated, and was described for the first time about 200 years ago. This conversion is most likely due to extended expression of a C class gene *AG* to the second organ whorl (Hintz *et al.*, 2006; Nutt *et al.*, 2006; Hameister *et al.*, 2013). *Capsella Spe* represents an exceptional (so-far known) case of a homeotic variant that has survived over 200 years in nature (Hameister *et al.*, 2013) suggesting that natural variation in homeotic genes has been maintained in nature and could become visible when temperature is increasing. Increased developmental variability under fluctuating temperatures may result in different floral phenotypes that perform better under different conditions. Over evolutionary timescales, such temperature-mediated responses in flower development may facilitate adaptation to local environments and contribute to diversification of floral traits.

### Future perspectives

Flowers are central for plant reproduction thus directly impacting fitness. Despite their key adaptive role, a knowledge gap remains regarding how environmental factors, particularly temperature, influence floral traits. Addressing this gap not only requires identification of the genetic and molecular factors underlying temperature effects on flower development, but also greater emphasis on exploiting natural variation and comparative approaches to uncover adaptive alleles in populations and their potential roles in responses to future temperatures.

In parallel, it is important to understand the contribution of phenotypic plasticity in temperature responses, particularly under ecologically realistic conditions. Future research should also focus on the combined effects of temperature with other abiotic factors, such as drought, elevated CO_2_, and changing light environments, to better reflect natural growth conditions.

Importantly, understanding the adaptive consequences of temperature-driven changes on flower development requires linking the molecular mechanisms of the temperature-mediated changes to fitness outcomes and plant–pollinator interactions. Finally, assessing how temperature-mediated effects in flowers will affect plant populations will demand integrative approaches that bridge molecular genetics, evolutionary biology, and ecology. Multi-trait and multi-environment experiments across diverse taxa will be essential to disentangle adaptive plasticity and to quantify the evolutionary potential of floral responses to climate change.

In addition to their fundamental role in plant adaptation and evolution, floral traits are central in horticulture and agriculture. In horticulture, artificial selection has been used to develop cultivars with larger and more visually appealing flowers. In agriculture, floral traits and inflorescence architecture are closely linked to yield and overall productivity, making them key targets for crop improvement. Consequently, identifying genetic loci underlying temperature resilience in floral development could inform breeding programmes aimed at stabilizing yield and reproductive success in crops under warming climates.

## Conclusion

While there are growing numbers of examples of changes in temperature affecting plant reproductive development, we are only starting to detail the genetic and molecular mechanisms underlying temperature-driven changes in flower development. Combining controlled genetic and molecular analyses with field-based ecological studies and predictive modelling will allow a more comprehensive understanding of how temperature shapes floral evolution.
